# Compatibilization of Polypropylene/Polyamide 6 Blend Fibers Using Photo-Oxidized Polypropylene

**DOI:** 10.3390/ma12010081

**Published:** 2018-12-27

**Authors:** Francesco Paolo La Mantia, Manuela Ceraulo, Maria Chiara Mistretta, Luigi Botta, Marco Morreale

**Affiliations:** 1 Department of Civil, Environmental, Aerospace, Materials Engineering, University of Palermo, Viale delle Scienze, 90128 Palermo, Italy; mariachiara.mistretta@unipa.it (M.C.M); luigi.botta@unipa.it (L.B.); 2 Department of Industrial and Digital Innovation, University of Palermo, Viale delle Scienze, 90128 Palermo, Italy; manuela.ceraulo@unipa.it; 3 Faculty of Engineering and Architecture, Kore University of Enna, Cittadella Universitaria, 94100 Enna, Italy; marco.morreale@unikore.it

**Keywords:** polymer blends, polypropylene, polyamide 6, compatibilization

## Abstract

The use of polyamide/polyolefin blends has gained importance and concern for years, but they also show some issues to be adequately addressed, such as the incompatibility between the two components. This is usually overcome by using suitable compatibilizers, typically based on functionalized polyolefins. However, there is only little information about the use of a degraded polyolefins to induce compatibilization. This is even truer, as far as polyamide 6/polypropylene (PA6/PP) blends are concerned. In this work, compatibilization of PA6/PP blends by using small amounts of photo-oxidized PP was investigated; furthermore, the effects due to the presence of the photo-oxidized PP were studied also in relationship to the spinning operation, where the existence of the non-isothermal elongational flow can lead to significant, further morphological changes. It was found that isotropic samples showed significant enhancements of the tensile properties upon adding the photo-oxidized PP. Under non-isothermal elongational flow conditions, the presence of the photo-oxidized PP was particularly effective in improving the mechanical properties in comparison to the uncompatibilized blend fibers. Furthermore, an important result was found: The elongational-flow processing allowed obtaining anisotropic samples where the improvements of the properties, in comparison to the isotropic samples, were similar to those achieved by using a compatibilizer.

## 1. Introduction

Polymer blends are a class of polymeric systems, which can allow obtaining “new” materials with a suitable range of properties, with a relatively good performance/cost ratio. Unfortunately, when chemically different polymers are blended, incompatibility leads to a final product with poor properties, often worse than those of the corresponding neat polymers [1,2,3,4,5,6].

Polyolefin/polyamide blends are of particular interest, since polyolefins provide better impact resistance at lower temperatures, as well as decent tensile strength, while polyamides provide oxygen barrier properties and higher elastic modulus; in particular, the most common polyolefin/polyamide blends are polyethylene/polyamide ones [5,6,7,8,9,10].

However, these blends are subject to strong incompatibility issues, which lead to very poor mechanical properties. Therefore, adequate compatibilization routes [9,11,12,13] must be undertaken in order to improve the properties of such systems, most of them relying on adding a suitable compatibilizer, often based on a polyolefin functionalized with polar groups, capable of reacting with the amine groups of the polyamide.

Further examples from literature can be cited such as, for instance, compatibilization of high density polyethylene (HDPE) or linear low density polyethylene (LLDPE) with polyamide 6 (PA6) by melt blending in the presence of HDPE or LLDPE previously functionalized by melt grafting with ricinoloxazoline maleinate monomer (two-step route), or in a one-step operation with simultaneous blending of the two (polar and non-polar) polymers with the monomer [7]. Rheological and mechanical tests pointed out positive effects by following the two-step route. Another example is based on reactive compatibilization of low-density polyethylene (LDPE) and polyamide 6 (PA6) with an ethylene-acrylic acid copolymer (EAA) and a low molar mass bis-oxazoline (PBO) [8]. It was confirmed by rheological, mechanical and morphological tests, that the compatibility of the LDPE/PA blend was significantly improved by the presence of the PBO, owing to the in-situ formation of EAA-grafted copolymers of both PE and PA.

Another example of polyolefin/polyamide blend that is also worth being taken into account is certainly represented by polypropylene /polyamide 6 (PP/PA6). PP has a suitable tensile strength for technological applications such as textiles and films, also providing low cost and dimensional stability (in presence of humidity) features, while PA6 can help in improving the tenacity and the elastic modulus, as well as oxygen barrier properties provided that, even in this case, suitable compatibilization is performed. Most compatibilization routes rely on functionalized PP, capable to react with the amine groups of the PA6 [9,11,12,14]. However, this route can be expensive since it needs the use of dedicated, chemically modified polypropylenes. Literature reports several studies about the compatibilization of PP/PA6 blends.

Miskolczi et al. [15] studied the compatibilization of PP/PA6 blends by patent-protected new polyalkenyl-poly-maleic-anhydride-based agents, finding significant improvements of tensile strength and elongation at break, which were partially explained on the basis of how the compatibilizers affected the PA crystallization.

Huber et al. [16] investigated the mechanical behavior of PA6/PP blends (90/10, 80/20, and 70/30 wt/wt), using two commercial maleic anhydride grafted PPs (MAPP) with a lower (2%) and a higher (7%) maleation degree, as compatibilizers. They found that the compatibilizer with a higher maleation degree showed a stronger reduction in interfacial tension, resulting in detrimental effects on the impact strength, although tensile strength improved similarly as in the case of a lower maleation degree compatibilizer.

Shin et al. [17] studied the properties of 80/20 PA6/PP blend compatibilized by electron-beam irradiation in the presence of a reactive agent. In particular, Glycidylmethacrylate (GMA) was used as a reactive agent for cross-copolymerization between PP and PA6 phase. It was found that the irradiation improved the compatibilization degree, on the basis of morphological observations and tensile strength results.

Other studies reported also the use of third components, such as fumed nanosilica [18], polyhedral oligomeric silsesquioxane (POSS) nanoparticles [19], room temperature ionic liquids (RTILs), and syntetic nanotalc [20].

However, there is only little information about the use of a degraded polyolefin to induce compatibilization. In the past, some of us [21,22] studied the properties of PA6/PE blends where PE was functionalized by photo-oxidation (either induced by accelerated or natural weathering). It was proved that the C=O groups, and in particular the carboxylic groups, can react with the amino end groups of the PA6, thus forming graft copolymers that can actually act as interfacial agents, resulting in blends with mechanical properties that are similar (and, in some cases even better) to those of blends with functionalized polyolefins. This idea can be, therefore, considered for application on PP/PA6 blends. Actually, to the best of our knowledge, only Jia et al. [23] investigated on compatibilization of recycled polypropylene in blends with polyamide 6. However, they focused on blends where the recycled polypropylene contained 21.5 wt % CaCO_3_ and came from waste household appliances, and the samples were prepared by injection molding.

In this work, therefore, we provide new information about compatibilization of PA6/PP blends by using small amounts of photo-oxidized PP and studying the related effects. In particular, the effect of the photo-oxidized PP is studied in relationship to the spinning operation, where the simultaneous presence of the non-isothermal elongational flow and a compatibilizer, can lead to significant morphological changes depending on both the flow and the compatibilizer used. A novel result is therefore found: The simultaneous presence of a photo-oxidized polyolefin and the elongational flow processing of PP/PA6 blends can be a promising, cost-effective, and environment-friendly approach for the optimization of the properties of such blends; moreover, it could be a starting point for investigations on more polymer blends, thus favoring new approaches, alternative to usual chemical compatibilization routes.

## 2. Materials and Methods

### 2.1. Materials and Sample Preparation

The polymers used in this work were a commercial polypropylene (PP) sample known as Capilene^®^ E50E (Carmel Olefins, Haifa, Israel, with a melt flow index of 1.8 g/10 min at 230 °C/2.16 kg) and a polyamide sample (PA6, Radilon S35 100 NAT, Radicinova, Gandino, Italy, with intrinsic viscosity in sulfuric acid = 3.4 dL/g).

Polypropylene sheets (about 200 micron thick) were subjected to accelerated weathering in a Q-UV (Q-Labs Corp., Westlake, OH, USA) chamber containing eight UVB-313 lamps. The exposure cycle conditions were: 8 h of light at T = 50 °C followed by 4 h water condensation at T = 40 °C. The PP samples (PPPh) were photo-oxidized for 72 h.

The preparation of the blends was carried out using a Brabender (Duisburg, Germany) Plasticorder PLE 300 batch mixer, at a temperature of 240 °C and a rotational speed of 60 rpm. The mixing time was about 10 min, enough to reach a constant value of the torque that means the attainment of thermo/fluidodynamic equilibrium. The blend composition was PA6/PP 80/20, wt/wt. The photo-oxidized PP (PPPh) amount was 5 wt % (wt % of the total). Some blends made of PPPh and PA6 were also prepared.

Sheets of all the samples were prepared by compression moulding at T = 240 °C (for approximately 3 min at a ~100 bar pressure) in a Carver (Wabash, IN, USA) press.

The fibres were spun using a capillary viscometer (Rheologic 1000, CEAST, Pianezza, Italy) operating under a constant extrusion speed (5 mm/min), with a 1 mm diameter (D_0_) die at 240 °C. The filaments were extruded in air, at room temperature. The take-up velocity was changed in order to obtain fibers with different draw ratios (DR). The draw ratio was calculated as:

DR = D_0_^2^/D_f_^2^ (1)

where D_0_ is the diameter of the capillary and D_f_ is the diameter of the fibres. In particular, DR = 2 up to DR = 10 were adopted in this work.

### 2.2. Characterization

The rheological characterization was carried out using the above-described capillary viscometer, operating at a temperature equal to 240 °C. In order to obtain some preliminary results over a wider shear rate range (i.e., including very low frequency values), some tests were carried out using a Mars III (Thermo Fisher, Waltham, MA, USA) plate-plate rotational rheometer. In general, all of the results showed good reproducibility (±5%).

Mechanical tests of the sheets and of the fibers were performed using an Instron (Norwood, MA, USA) mode. 3365 universal testing machine, operating at 1 mm/min speed, with an increase to 100 mm/min after 10% deformation was achieved, up to final breaking. The values of the elastic modulus, E, the tensile strength, TS, and the elongation at break, EB, were calculated as average of at least five tests, with adequate reproducibility (±8%).

The morphology of the blends was studied via scanning electron microscopy (SEM). SEM micrographs were obtained on samples fractured in liquid nitrogen and gold- sputtered (in order to make them conductive), using a FEI (Hillsboro, OR, USA) Quanta 200F scanning electron microscope.

Fourier-Transform Infra-Red (FT-IR) spectra were collected by means of a Perkin-Elmer (Waltham, MA, USA) Spectrum One spectrometer. Spectra were measured with 8 scans and a 4 cm^−1^ resolution. In order to quantitatively compare the spectra of the blends, we considered normalization over the vibration band of –CH_2_– groups (1462 cm^−1^) [24]. The carbonyl concentration was evaluated by considering the peak at 1718 cm^−1^ and an extinction coefficient of 350 L mol^−1^ cm^−1^ [25]. 

Molau tests [26,27,28] were carried out by dissolving 200 mg of the sample in 10 mL of 80% (v/v) formic acid.

## 3. Results

### 3.1. Characterization of the Photooxidized PP

**Figure 1:**
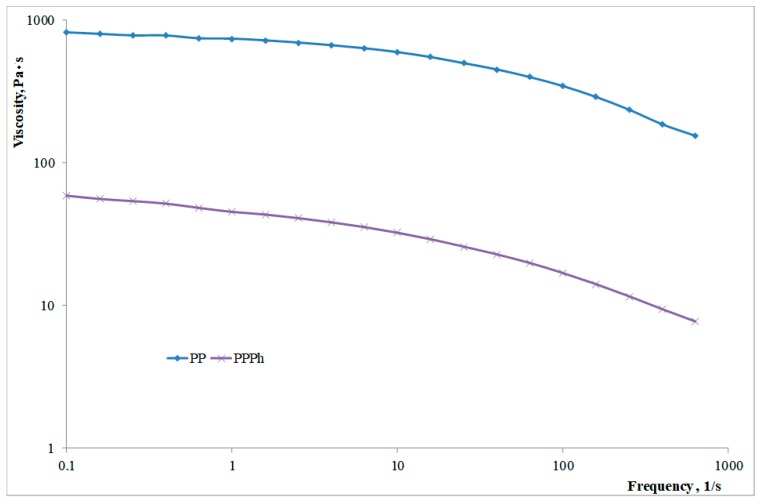
Flow curves of virgin and photo-oxidized PP.

In Figure 1, the flow curves of virgin and photo-oxidized PP are reported. The dramatic decrease of the Newtonian viscosity is a clear evidence of the degradation phenomena undergone by the polypropylene sample during the accelerated photo-oxidation step.

The decrease of the molecular weight can be evaluated considering that the Newtonian viscosity depends on the molecular weight through the well-known relationship:

η_0_ = K·M_w_^3.4^ (2)

where η_0_ is the Newtonian viscosity, K is a constant which depends on the polymer and on the temperature.

It is possible to evaluate the value of the Newtonian viscosity by using the Ferry’s equation:

1/η = 1/η_0_ + bτ (3)

where τ is the shear stress, and η_0_ and η the Newtonian viscosity and the viscosity at a given value of the shear stress, respectively [29].

The decrease of the molecular weight is then evaluated as

M_w_(PPPh)/M_w_(PP) = (η_0_(PPPh)/η_0_(PP))^1/3.4^ (4)

It was therefore found that the molecular weight undergoes a decrease of approximately 58%.

**Figure 2:**
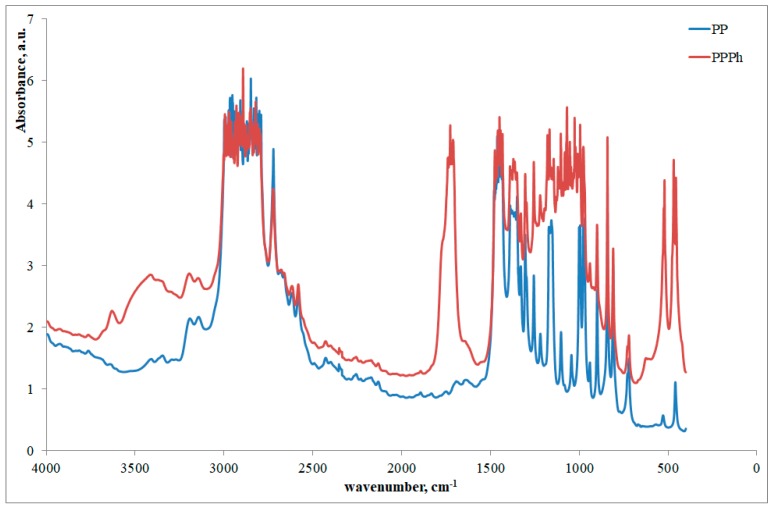
FTIR spectra of virgin and photo-oxidized PP.

The effects of the photo-oxidative degradation are also well evidenced by the FTIR spectra reported in Figure 2.

A remarkable increase of the band of the carbonyl and of the hydroxyl points out the relevant photo-oxidation undergone by the PP. This was further proved by calculation the carbonyl concentration, which was found to be (obviously) 0 in the neat PP, and 0.54 mol/L in the photo-oxidized PP.

In Table 1, the mechanical properties of the isotropic sheets of virgin and photo-oxidized PP are reported.

The degradation phenomena had an obvious impact on the tensile properties. More in details, the embrittlement undergone because of the degradation leads to an increase of the elastic modulus and, especially, a dramatic decrease of the elongation at break and of the tensile strength. This was in agreement with our previous studies on photo-oxidation of neat PP [30,31].

### 3.2. Isotropic Blends

**Figure 3:**
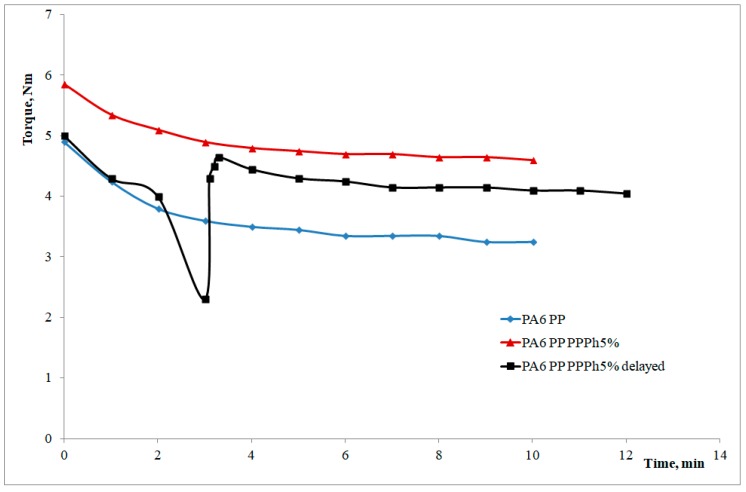
Torque curves as a function of the mixing time.

In Figure 3, the torque curves as a function of the mixing time of the blends having the same composition and different mixing procedures are reported.

The curve of the PA6/PP blend (i.e., containing only virgin PP) is lower than that of the blend containing 5 wt % photo-oxidized polymer. This means that the viscosity of this latter blend is higher than that of the blend with the virgin PP and the interpretation of this behaviour should be related to some reactions between the –COOH groups of the PPPH and the amine groups of the polyamide during mixing, which gives rise to new macromolecules that, in turn, can act as compatibilizers between the two components. This was further confirmed by examining the torque curves of a blend prepared with the two components (PA6 and virgin PP) and a subsequent addition of the photo-oxidized PP only after two minutes of mixing. As can be observed, the curve decreases during the addition due to the very low viscosity of the photo-oxidized PP, and then rises due to of the reactions between the PPPh and the PA6 which, improving the adhesion between the two phases, increases the overall viscosity of the blend. The small difference in the final value of the torque between the two systems containing PPPh can be attributed to some small differences in the molecular weight and in the C=O groups present in the PPPh samples actually used in these tests.

**Figure 4:**
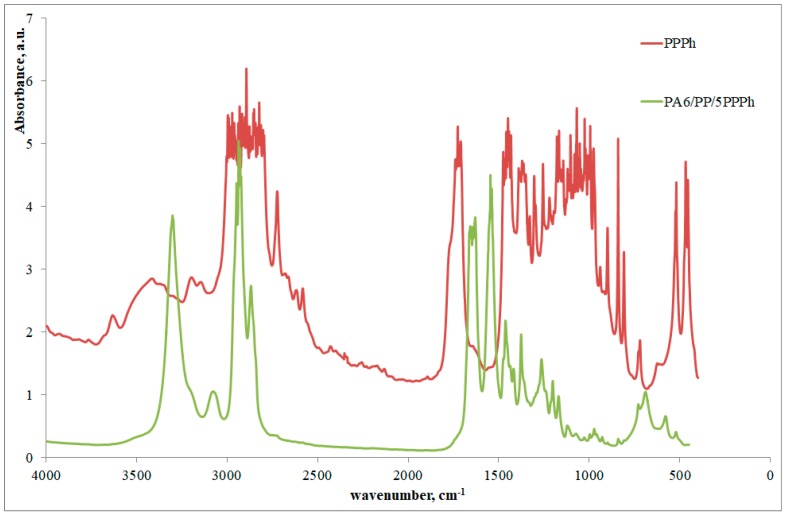
FTIR spectra of photo-oxidized PP (PPPh) and PA6/PP/PPPh blend.

Furthermore, FTIR spectra of the PA6/PP/PPPh blend were taken and shown in Figure 4. It is observed that the –COOH group peak, present in the PPPh spectra, is not present in the PA6/PP/PPPh blend, reasonably due its reaction with the amine groups of the PA6, in agreement with the previously written hypotheses.

**Figure 5:**
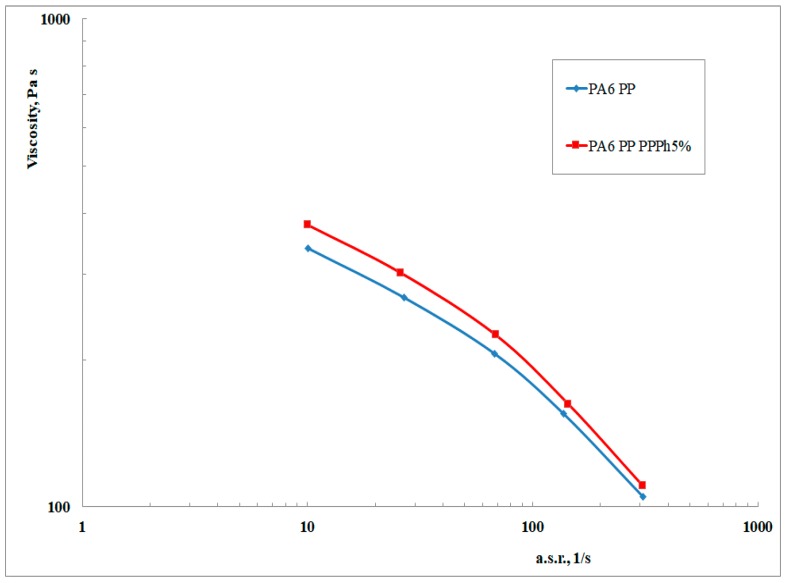
Flow curves obtained in the capillary viscometer of the binary and ternary blends.

In Figure 5, the flow curves of the uncompatibilized and compatibilized blends are reported. It can be noticed that the ternary blend shows a higher viscosity in comparison to the binary blend. This is in agreement with what could be expected from the previously discussed torque values, and therefore further confirms, over a wide range of shear rates (and thus, of shear stresses), that compatibilizing macromolecules are formed by reactive mixing. Of course, the differences are not dramatic since the measurement is performed at relatively high shear rates, where they typically tend to decrease. However, such shear rates are of greater interest since it is comparable to those usually attained during industrial (e.g., injection molding) operations.

In Table 2, the mechanical properties (elastic modulus, E, tensile strength, TS, elongation at break, EB) of isotropic sheets of the uncompatibilized and compatibilized blend are reported.

The increase of all the mechanical properties confirm the compatibilizing action of the PPPh. In particular, it is worth noting that the elastic modulus increases of 57%, the tensile strength of 68%, and the elongation at break of 22%.

**Figure 6:**
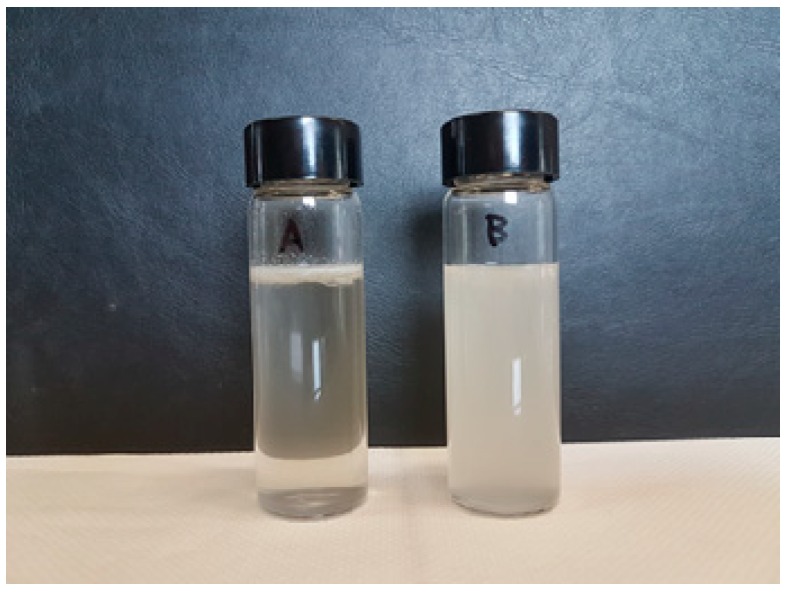
Molau tests on the PA6/PP blend (**A**) and the PA6/PP/PPPh blend (**B**).

In Figure 6, the photographs from the Molau tests on binary (A, left) and ternary (B, right) blends are shown. It can be observed that the solution is clear in the case of the binary blend, and it consists in PA6 (soluble in formic acid), while the supernatant layer is a suspension of PP particles (insoluble in formic acid). On the other hand, the ternary blend leads to an evident and persistent turbidity, representing a suspension of colloidal particles. It is known [26,27,28] that this colloidal suspension should be attributed to the existence of PP/PA6 graft copolymers, here behaving like interfacial agents. These could be attributed to the previously discussed reactions between the –COOH groups of the PPPh and the amine groups of the polyamide during mixing.

**Figure 7:**
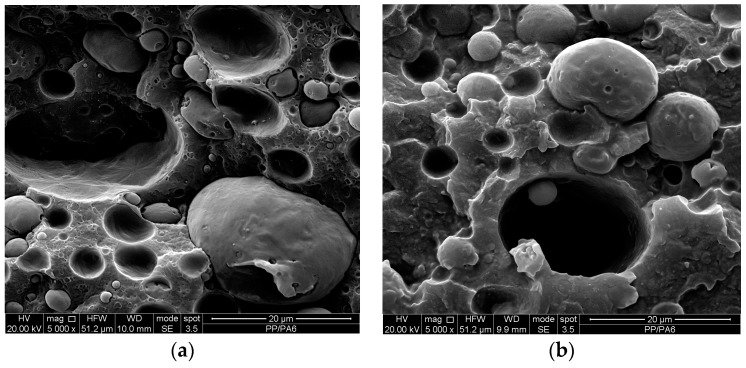

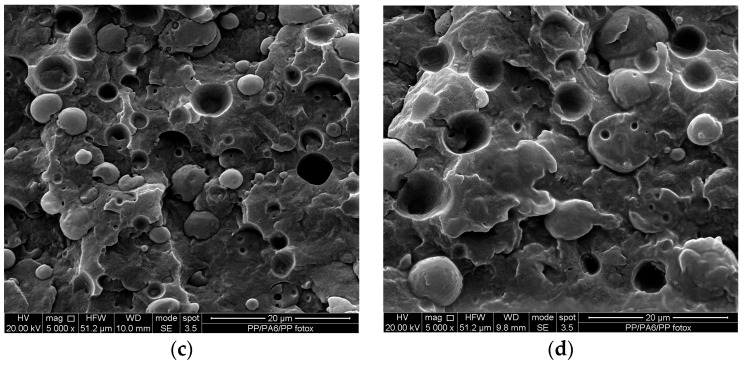
SEM micrographs of the binary (**a**,**b**) and ternary (**c**,**d**) blends.

The SEM micrographs of the sheets of the two blends are reported in Figure 7. The binary blend shows the usual picture of incompatible blend: The particles of the dispersed phase show heterogeneous dimensions and are not adherent to the continuous phase. The same particles of the dispersed phase show, on the contrary, a narrower dispersion of the dimensions and are much more adherent to the matrix. The macromolecules coming from the reaction between polyamide and PPPh act as a bridge between the two phases, thus decreasing the interfacial tension between the two components and improving the adhesion between the two phases.

### 3.3. Characterization of Anisotropic Blends

**Figure 8:**
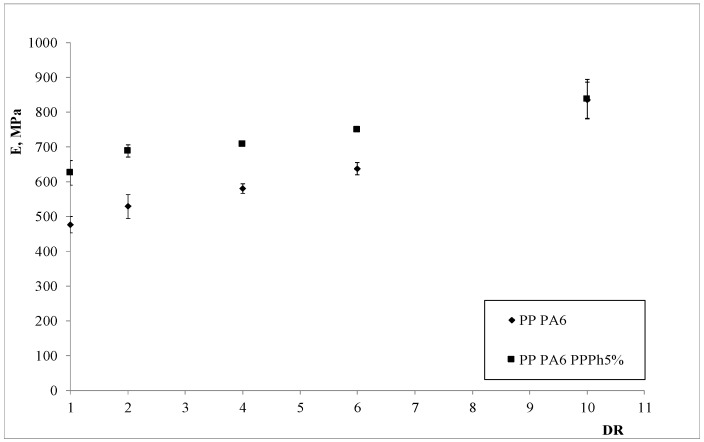
Elastic modulus as a function of the draw ratio of the two blends.

**Figure 9:**
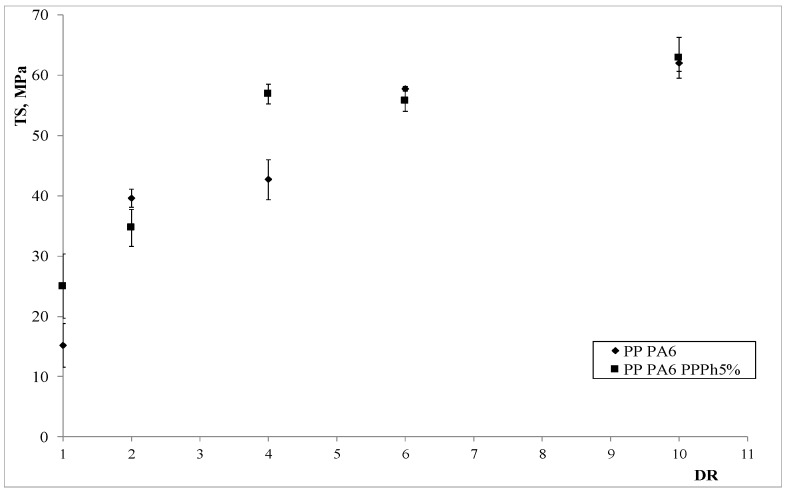
Tensile strength as a function of the draw ratio of the two blends.

**Figure 10:**
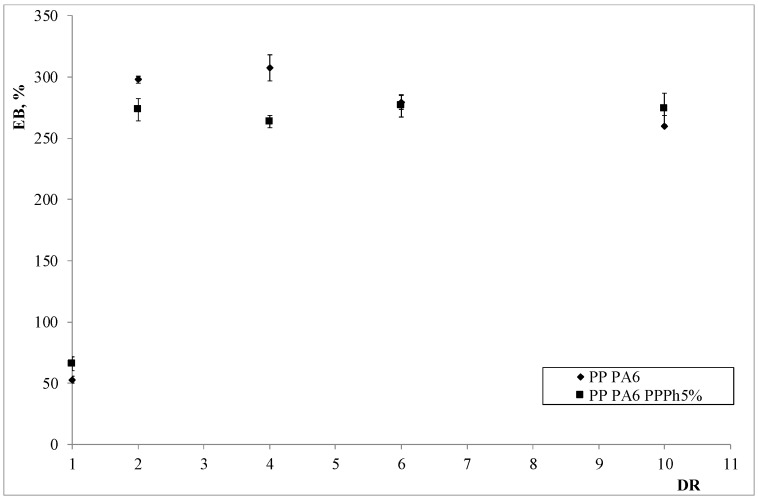
Elongation at break as a function of the draw ratio of the two blends.

The mechanical properties of the anisotropic blends are reported in Figure 8, Figure 9 and Figure 10.

The observation of the plots showing the trends of the tensile properties allows drawing some interesting considerations. The elastic modulus and the tensile strength experience significant enhancements on increasing the DR; however, the values of the elongation at break, although varying marginally upon increasing the DR, are dramatically higher than those observed in the corresponding, un-oriented (isotropic) samples. Therefore, a very interesting result is found: The processing under non-isothermal elongational flow conditions can lead to final results which are similar to those obtainable by means of chemical compatibilization (which typically leads to an improvement of the elongation at break [32]). This provides a further explanation of the better mechanical properties found, and is in agreement with the results we recently found in a LDPE/PA6 system [32]. Furthermore, the presence of the PPPh leads to some improvement of the properties, especially with concern to the elastic modulus and the elongation at break, where such enhancements are quite significant. This is a confirmation of the compatibilizing effect of the photo-oxidized polypropylene.

**Figure 11:**
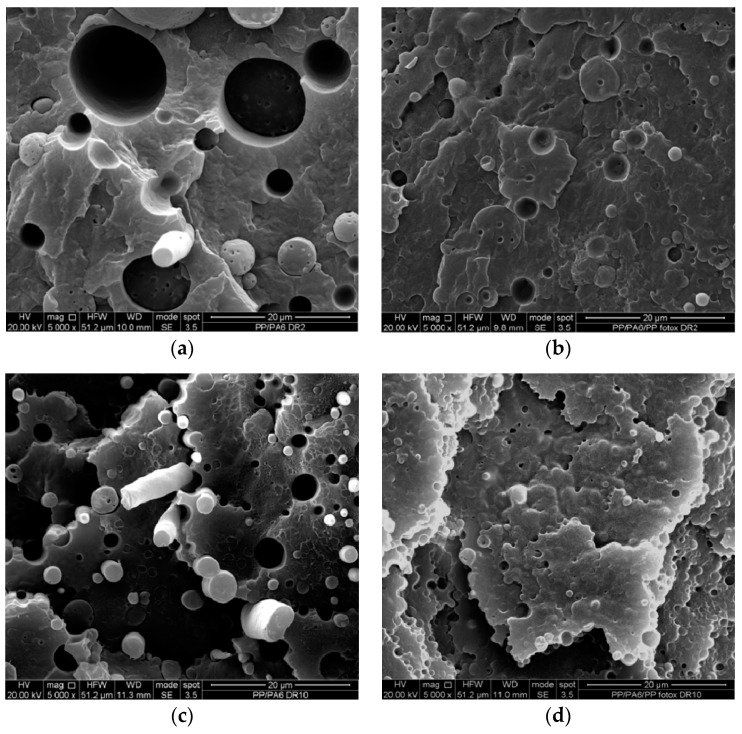
SEM micrographs of the binary (**a**,**c**) and ternary (**b**,**d**) anisotropic blends. (**a**,**b**) refer to anisotropic samples obtained with DR = 2, while (**c**,**d**) to samples with DR = 10.

Figure 11a–d reports the SEM micrographs of both binary and ternary anisotropic samples obtained at different draw ratios. First considerations can be drawn with regard to the effect of the photo-oxidized PP, comparing Figure 11a with Figure 11b, and Figure 11c with Figure 11d. It can be clearly observed that the presence of the photo-oxidized PP leads to a reduction in the dimension of the second-phase domains and of the voids, with the interface surfaces (here easily observable as the internal surfaces of the voids) being less sharp in the ternary blends. This clearly points out that the photo-oxidized PP actually acted as a compatibilizer.

On the other hand, the direct comparison between Figure 11a with Figure 11c, as well as for Figure 11b with Figure 11d, allows outlining additional information, i.e., that the morphology of the samples improves by increasing the DR, and moreover, that the dispersed-phase particles undergo a remarkable orientation. This, in turn, has clearly some positive effects on the values of the elastic modulus and the tensile strength, while the elongation at break experiences lower variations; once more, the presence of the PPPh improves the interfacial behaviour, since its presence allows keeping practically constant values on increasing the DR, while the uncompatibilized blend experiences significant reductions at higher DRs.

As regards the comparison between the oriented (anisotropic) and the unoriented (isotropic) blends (Figure 11 vs. Figure 7, respectively), it can be once more observed that the morphology experiences some improvements, with the dispersed phase particles having smaller size and clearly showing orientation, in agreement with the improvements found from the tensile tests. In conclusion, the processing under non-isothermal elongational flow conditions lead to final effects that are similar to those obtainable by means of chemical compatibilization, and this is in agreement with the findings we have recently discovered in an LDPE/PA6 system [32].

## 4. Conclusions

In this work, the validity of PA6/PP blends compatibilization by using small amounts of photo-oxidized PP was investigated. It was found that isotropic samples experienced significant improvements of the tensile properties upon adding 5 wt.% photo-oxidized PP. This was attributed to effective reactions between the carbonyl groups contained in the photo-oxidized PP, and the amino groups of the PA6, and was further proved by torque measurements and rheological characterization.

Then, the effect of the photo-oxidized PP was studied in presence of the non-isothermal elongational flow induced by spinning operation. It was observed that the presence of the photo-oxidized PP is significantly effective in improving the mechanical properties of the ternary-blend fibers in comparison to binary-blend ones; furthermore, a very interesting result was found, i.e., the elongational-flow processing allowed obtaining anisotropic samples where the enhancements of the properties, in comparison to isotropic samples, were similar to those achieved by using a compatibilizer.

## Figures and Tables

**Table 1:** Mechanical properties of virgin and photo-oxidized PP.

Sample	E (MPa)	TS (MPa)	EB (%)
PP	465	19	467
PPPh	763	4	1

**Table 2:** Mechanical properties of the PP/PA6 uncompatibilized and compatibilized blend.

Sample	E (MPa)	TS (MPa)	EB (%)
PP/PA6	460	12.6	51
PP/PA6/PPPh	722	21.2	62
